# An outbreak of extremely drug-resistant *Pseudomonas aeruginosa*in a tertiary care pediatric hospital in Italy

**DOI:** 10.1186/1471-2334-14-494

**Published:** 2014-09-10

**Authors:** Marta Ciofi degli Atti, Paola Bernaschi, Michaela Carletti, Ida Luzzi, Aurora García-Fernández, Alice Bertaina, Annamaria Sisto, Franco Locatelli, Massimiliano Raponi

**Affiliations:** Unit of Clinical Epidemiology, Medical Direction, Bambino Gesù Children’s Hospital, Piazza S. Onofrio, 4, Rome, 00161 Italy; Unit of Microbiology, Bambino Gesù Children’s Hospital, Rome, Italy; Department of Infectious, Parasitic and Immunomediate Diseases, Istituto Superiore di Sanità, Rome, Italy; Department of Hematology/Oncology, Bambino Gesù Children’s Hospital, Rome, Italy; Medical Direction, Bambino Gesù Children’s Hospital, Rome, Italy

**Keywords:** Pseudomonas aeruginosa, Extensively-drug resistance, Healthcare-associated infections, Children

## Abstract

**Background:**

Extensively drug-resistant *Pseudomonas aeruginosa* (XDR-PA) isolates are susceptible to only one or two classes of antibiotics. In 2011–2012, we investigated an outbreak of XDR-PA affecting children with onco-hematological diseases.

**Methods:**

Outbreak investigation included ascertainment of cases, tracing of intestinal carriers and environmental surveillance. Contact precautions were adopted for patients with infection or colonization. Isolates were tested for antimicrobial susceptibility; phenotypic confirmation of carbapenemase production was performed, and carbapenemase genes were tested by multiplex polymerase-chain-reaction (PCR). Genotypes were determined by pulsed-field gel electrophoresis (PFGE).

**Results:**

XDR-PA was isolated from 27 patients; 12 had bacteremia, 6 other infections and 9 were colonized. Severe neutropenia was significantly associated with bacteremia. Bloodstream-infection mortality rate was 67%. All isolates were resistant to carbapenems, cephalosporins and penicillins + *β*-lactamase inhibitors. Isolates were susceptible only to colistin in 22 patients, to colistin and amikacin in 4, and to ciprofloxacin and colistin in 1. PFGE results identified 6 subtypes of a single genotype, associated with clusters of cases, and 4 sporadic genotypes. Two sporadic isolates were metallo-β-lactamase producers, negative to PCR. All other isolates were metallo-β-lactamase producers due to the presence of a VIM carbapenemase. Incidence of XDR-PA infections decreased from 0.72 cases/1,000 inpatient-days in March 2011-March 2012, to 0.34/1,000 in April-December 2012, after implementation of active finding of intestinal carriers on all onco-hematological inpatients.

**Conclusions:**

Control measures targeting intestinal carriers are crucial in limiting in-hospital transmission of XDR-PA polyclonal strains, protecting more vulnerable patients, such as severely neutropenic children, from developing clinical infections.

**Electronic supplementary material:**

The online version of this article (doi:10.1186/1471-2334-14-494) contains supplementary material, which is available to authorized users.

## Background

*Pseudomonas aeruginosa* is one of the leading causes of nosocomial bloodstream infections and pneumonia [1, 2]. The primary site of colonization and a frequent source of subsequent infection by *P. aeruginosa* is the gastrointestinal tract, where as many as 50% of critically ill patients are found to be colonized within 3 days of admission with as many as 30% of strains displaying antibiotic resistance [3].

Several *P. aeruginosa* nosocomial outbreaks caused by patient-to-patient transmission, environmental sources or contaminated medical devices have been described [4–6]. Over recent years, nosocomial infections caused by multi-drug-resistant *P. aeruginosa* (MDR-PA) have been reported in adults and children [7–11]. Multi-drug resistance is defined as non-susceptibility to at least one agent in three or more antimicrobial categories. Extensively drug-resistant (XDR) bacterial isolates remain susceptible to only one or two classes of antimicrobials [12]. To date, XDR *P. aeruginosa* (XDR-PA) nosocomial outbreaks have been described in adults [13, 14]. In this article, we report and characterize an XDR-PA outbreak in a tertiary-care pediatric hospital in Italy.

## Methods

### Setting

The Bambino Gesù Children’s Hospital is a tertiary care hospital in Rome, Italy, with 607 inpatient beds. In 2011, hospital acute inpatient admissions were 24,449. Hospital patient population includes children at high risk of acquiring healthcare-associated infections (HAI), such as pre-term newborns and immunocompromised patients. In-hospital actions for preventing and controlling HAI have been implemented over time [15], and in the years 2007–2010 the annual point prevalence of HAI significantly decreased from 7.6% to 4.3% (p < 0.001) [15]. In 2011, HAI annual point prevalence was 3.4% (unpublished data). At that time, no active surveillance of MDR Gram negative intestinal carriers was in place. The Department of Pediatric Hematology/Oncology includes several wards for inpatient hospitalization, with a total of 54 inpatient beds, and one outpatient clinic.

### Case definitions

Patients who had XDR-PA cultured from blood and no evident site of infection were defined as bacteremia cases. Other infections caused by XDR-PA were defined according to presence of signs and symptoms, and site of isolation [16]. Patients with positive clinical samples from non-sterile sites without related signs or symptoms of infection were defined as colonized.

### Case finding

Microbiological Laboratory results were retrospectively reviewed to verify if there were patients with XDR-PA strains isolated prior to September 2011. Since September 2011, the Microbiology Laboratory transmitted by e-mail to Infection Control Team (ICT) information on all patients with XDR-PA isolates (patient demographics, ward of hospitalization, type of biological sample, date of sample collection). ICT reviewed medical records for patient’s clinical data (reason for hospital admission, underlying diseases, signs and symptoms related to XDR-PA infection and their date of onset, in-hospital patient transfers, status at hospital discharge). Since October 2011, active tracing of intestinal carriers was implemented among patients hospitalized in the same ward and period of time as a patient with bacteremia or other infections due to XDR-PA. In March 2012, active tracing of intestinal carriers was extended to all inpatients admitted to onco-hematology wards. Stool samples were collected at admission and once weekly until discharge.

### Environmental surveillance

Environmental sampling was performed throughout the outbreak period. Sterile cotton swabs were used to obtain samples from water outlets, sinks, drains, beds and surfaces in patient rooms, and surfaces of nurse’s stations. Samples of tap water were also obtained.

### Control measures

Outbreak control measures were based on intensifying contact precautions with patients with infection or colonization. Contact precautions required health care workers to wear a gown and gloves for all interactions that might involve contact with the patient or potentially contaminated areas in the patient’s environment, wearing personal protective equipments upon entry in the room and discarding them before exiting the patient room. Adherence to antiseptic hand hygiene was also reinforced, along with cleaning of patient rooms. Hospitalized patients were isolated or cohorted; if this was not possible, a ≥1 meter spatial separation between beds was requested. Rooms hosting patients subjected to contact precautions were identified with an alert poster; parents and caregivers were educated to comply with contact precautions. Implementation of contact precautions, including identification of patient rooms and documentation of parents/caregivers education on patient clinical record was actively verified by ICT. Precautions were maintained until the patient had three cultures negative for XDR-PA, or until hospital discharge. Information on carriage was reported on hospital discharge letter. Contact precautions were adopted during outpatient visits of children who were colonized.

### Microbiological and molecular biology studies

*P. aeruginosa* was identified and tested for antimicrobial susceptibility by Vitek 2 automated systems (bioMérieux, Marcy l’Etoile, France) using AST-N201 and AST-N203 Gram Negative Susceptibility Card. On the basis of their resistance phenotype, all *P. aeruginosa* strains were further tested, to confirm resistance, with E-Test quantitative method for the following antimicrobial agents: amikacin, gentamicin, ceftazidime, ciprofloxacin, colistin, meropenem, piperacillin/tazobactam, tobramycin. Results were interpreted according to breakpoint criteria defined by the European Committee on Antimicrobial Susceptibility Testing (EUCAST) for clinical susceptibility characterization [17]. Phenotypic confirmation of the carbapenemase production was tested by combined-disc method using a disc of meropenem (10 μg) (Becton Dickinson, Milan, Italy) with and without 400 μg phenylboronic acid and with a disc of meropenem (10 μg) with and without 10 μL 0.1 M EDTA on Mueller-Hinton agar II (Oxoid, Basingstoke, UK) [18]. The carbapenemase production was also tested by using agar tablet/disc diffusion method by the KPC/MBL and OXA, 48 Confirm Kit (ROSCO Diagnostica A/S, Taastrup, Denmark). The presence of carbapenemase genes (*bla*_VIM_, *bla*_IMP_, *bla*_OXA-48_, *bla*_NDM_, *bla*_KPC_, *bla*_SPM_, *bla*_BIC_, *bla*_SIM_, *bla*_GIM_, *bla*_DIM_, *bla*_AIM_) was confirmed by multiplex Polymerase-chain-reaction (PCR) [19].

To investigate the genetic relationship among the isolates, XDR-PA strains were analyzed by Pulsed-Field Gel Electrophoresis (PFGE), performed according to the PulseNet standardised protocol [20], using XbaI as the restriction enzyme (New England Biolabs, Ipswich, MA). To avoid degradation of DNA samples, 50 μM thiourea was added to the running buffer and agarose gel [21]. *Salmonella enterica* serotype Braenderup H9812 strain was used as molecular size marker [22]. Dendrogram and cluster analysis were performed using algorithms available in the BioNumerics software package v.6.0 (Applied Maths, Sint-Martens-Latem, Belgium). Percent similarity between different chromosomal fingerprints was scored by the Dice coefficient. The unweighted pair group method with arithmetic means (UPGMA), with a 1.00% tolerance limit and 1.00% optimization, was used to obtain the dendrogram. Isolates with PFGE patterns differing in one to four restriction fragments (coefficient of similarity ≥ 90%) were considered to belong to the same genotype. Subtypes were assigned to isolates differing by one to four bands (coefficient of similarity ≥ 95%) [23].

### Ethical considerations

All the laboratory investigations performed to patients were part of standard care for outbreak investigation and control. All the data were collected for management of the outbreak, in accordance with the hospital policy regarding outbreak investigations approved by the infection control committee. For these reasons, the approval of the ethical committee was not deemed necessary.

## Results

From March 2011 to December 2012, 27 isolates of XDR-PA were collected from 27 patients with infection or colonization. Most patients (20/27; 74.0%) had a diagnosis of either acute leukemia or lymphoma; their median age was 12 years (range: 1–27 years), and 59% were female (Table 1). Among them, there were 12 patients with bacteremia, 6 patients with other infections and 9 patients who were colonized. Profound neutropenia, defined as <100 white blood cells/μL (WBC/μL), was significantly more frequent among patients with bacteremia than among patients with other types of infection or colonization (83% vs 13%; p < 0.001). Eight of the 12 patients with bacteremia died because of XDR-PA bloodstream infection (67%). No deaths related to XDR-PA or within 30 days from laboratory confirmation occurred in patients with other sites of infection or colonization.

**Table 1:** **Characteristics of patients with infections or colonization due to XDR** ***P. aeruginosa*** **; Bambino Gesù Children’s Hospital, 2011–2012 (N: 27)**

	Bacteremia	Other infections	Colonization	Total
Number of patients	12	6	9	27
	N (%)	N (%)	N (%)	N (%)
Age				
*Median*	11.5	15	11	12
*Range*	(2;24)	(3;27)	(1;16)	(1;27)
Gender				
*Male*	4 (33.3%)	2 (33.3%)	5 (55.6%)	11 (40.7%)
*Female*	8 (66.7%)	4 (66.6%)	4 (44.4%)	16 (59.3%)
Diagnosis				
*Leukemia/lymphoma*	11 (91.7%)	4 (66.6%)	5 (55.6%)	20 (74.1%)
*Solid tumor*	0	0	1 (11.1%)	1 (3.7%)
*Marrow failure*	0	1 (16.6%)	0	1 (3.7%)
*Inborn error*	1 (8.3%)	1 (16.6%)	3 (33.3%)	5 (18.5%)
Chemotherapy				
*Yes*	8 (66.7%)	1 (16.6%)	3 (33.3%)	12 (44.4%)
*No*	4 (33.3%)	5 (83.3%)	6 (66.7%)	15 (55.6%)
HSCT				
*Yes*	4 (33.3%)	1 (16.6%)	1 (11.1%)	6 (22.2%)
*No*	8 (66.7%)	5 (83.3%)	8 (88.9%)	21 (77.8%)
Neutropenia (severity)				
*<100 WBC/mm* ^*3*^	10 (83.3%)	1 (16.6%)	1 (11.1%)	12 (44.4%)
*100-500 WBC/mm* ^*3*^	2 (16.7%)	0	0	2 (7.4%)
*>500-1000 WBC/mm* ^*3*^	0	0	1 (11.1%)	1 (3.7%)
*>1000 WBC/mm* ^*3*^	0	5 (83.3%)	7 (77.8%)	12 (44.4%)
Outcome				
Died	8 (66.7%)	0	0	8 (29.6%)
Alive	4 (33.3%)	6 (100%)	9 (100%)	19 (70.4%)

WBC: white blood cells; HSCT: Hematopoietic stem cell transplantation.

All isolates from patients with infection or colonization were resistant to carbapenems, cephalosporins, and penicillins + β-lactamase inhibitors. Isolates from 22 patients were susceptible only to colistin; isolates were susceptible to colistin and amikacin in 4 patients, and susceptible to ciprofloxacin and colistin in 1.

The first in-hospital case occurred in March 2011, in a 4-year-old Italian child affected by acute lymphoblastic leukemia who had a genital lesion from which XDR-PA was cultured. Thirteen of the 18 patients (72%) with bacteremia or infections other than bacteremia occurred between July 2011 and March 2012, while the majority of colonized patients were identified from March to November 2012 (6/9; 67%). Due to their underlying disease, all patients were admitted to the hospital several times, either for inpatient care or as outpatients. After the index case, an epidemiological link was documented for 23/26 patients, who were visited as outpatients on the same day (2 patients) or hospitalized in the same ward and period of time (21 patients). All inpatient wards were involved.

**Figure 1:**
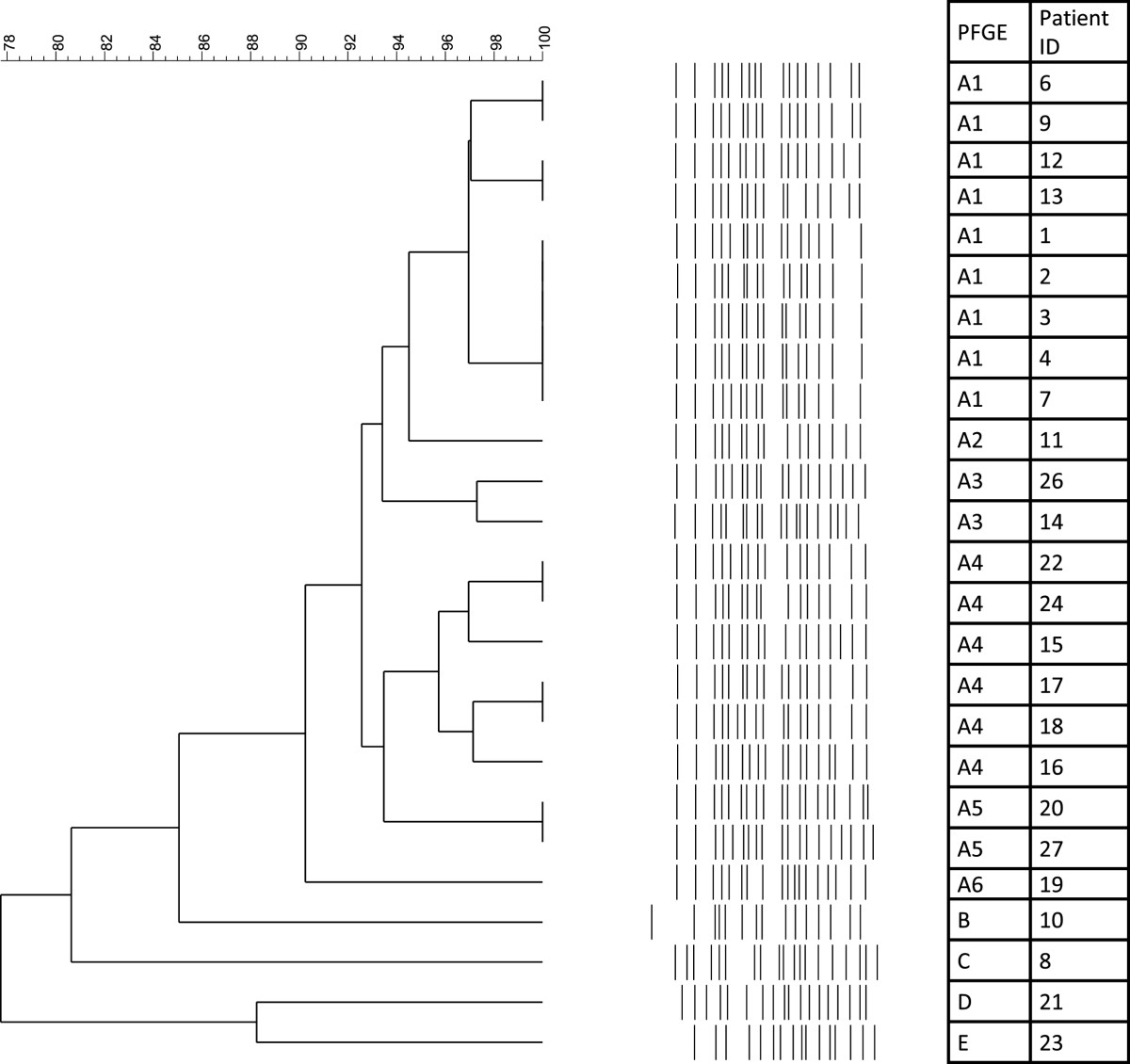
**PFGE analysis of** ***P. aeruginosa*** **isolates.** Bambino Gesù Children’s Hospital, 2011–2012 (N: 25).

**Figure 2:**
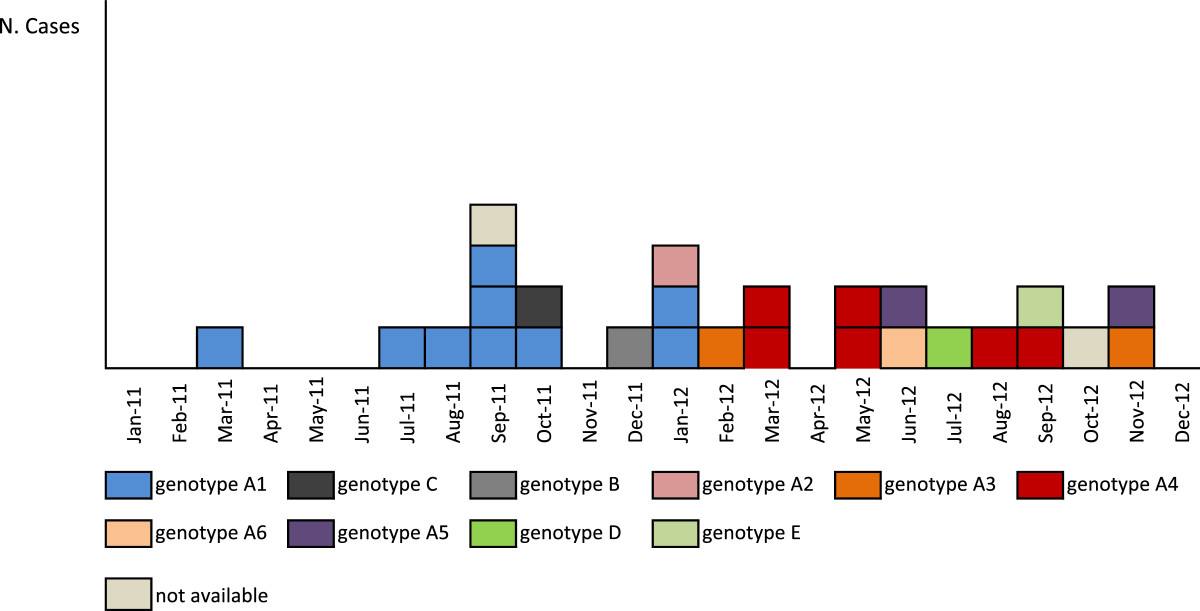
**Patients with XDR** ***P. aeruginosa*** **infection or colonization by month of laboratory confirmation and genotype.** Bambino Gesù Children’s Hospital, 2011-2012 (N: 27).

Incidence of XDR-PA infections decreased from 0.72/1,000 inpatient days (13 cases; 18,030 inpatient days) in the period March 2011-March 2012, to 0.34/1,000 in April-December 2012 (5 cases; 14,839 inpatient days) after implementation of active surveillance of intestinal carriage among all inpatients hospitalized in the onco-hematology wards.PFGE was performed on 25 isolates from 25 different patients; isolates from the two remaining patients were not available for PFGE testing. Results showed that the 25 isolates belonged to 5 different genotypes (A-E) (Figure 1).A high degree of genetic similarity (≥95%) was observed within and between 6 pattern (A1-A6) in cluster A. Subtype A1 was the most predominant pattern including 9 strains isolated from March 2011 to January 2012 (Figure 2). Subtype A4 was isolated from March to September 2012 from 6 patients. Subtypes A2, A3, A5 and A6 were isolated from one or two patients. The remaining 4 isolates (B-E) had different genotypes and were sporadic. The proportion of cases with bacteremia and the bloodstream infections mortality rate did not significantly vary by genotype.

The carbapenemase phenotypic tests on the 25 available isolates showed that all isolates presented a metallo-β-lactamase (MBL)-production phenotype. This phenotype was confirmed by carbapenemase multiplex PCR in all genotypes, except C and E, which were negative by multiplex PCR (Table 2).

**Table 2:** **Characteristics of XDR** ***P. aeruginosa*** **isolates; Bambino Gesù Children’s Hospital, 2011–2012 (N: 27)**

Patient Id.	Month/Year of isolation	Site of isolation	PFGE	Phenotyping	Carbapenemases multiplex PCR
1	03/2011	Vaginal swab	A1	Metallo-β-lactamase	*bla*_VIM_
2	07/2011	Blood	A1	Metallo-β-lactamase	*bla*_VIM_
3	08/2011	Blood	A1	Metallo-β-lactamase	*bla*_VIM_
4	09/2011	Blood	A1	Metallo-β-lactamase	*bla*_VIM_
5	09/2011	Blood	Not available		
6	09/2011	Blood	A1	Metallo-β-lactamase	*bla*_VIM_
7	09/2011	Urine	A1	Metallo-β-lactamase	*bla*_VIM_
8	10/2011	Surgical site	C	Metallo-β-lactamase	Negative
9	10/2011	Stool	A1	Metallo-β-lactamase	*bla*_VIM_
10	12/2011	Blood	B	Metallo-β-lactamase	*bla*_VIM_
11	01/2012	Urine	A2	Metallo-β-lactamase	*bla*_VIM_
12	01/2012	Vaginal swab	A1	Metallo-β-lactamase	*bla*_VIM_
13	01/2012	Ocular secretion	A1	Metallo-β-lactamase	*bla*_VIM_
14	02/2012	Blood	A3	Metallo-β-lactamase	*bla*_VIM_
15	03/2012	Blood	A4	Metallo-β-lactamase	*bla*_VIM_
16	03/2012	Stool	A4	Metallo-β-lactamase	*bla*_VIM_
17	05/2012	Blood	A4	Metallo-β-lactamase	*bla*_VIM_
18	05/2012	Blood	A4	Metallo-β-lactamase	*bla*_VIM_
19	06/2012	Blood	A6	Metallo-β-lactamase	*bla*_VIM_
20	06/2012	Surgical site	A5	Metallo-β-lactamase	*bla*_VIM_
21	07/2012	Stool	D	Metallo-β-lactamase	*bla*_VIM_
22	08/2012	Stool	A4	Metallo-β-lactamase	*bla*_VIM_
23	09/2012	Tracheal aspirate	E	Metallo-β-lactamase	Negative
24	09/2012	Blood	A4	Metallo-β-lactamase	*bla*_VIM_
25	10/2012	Urine	Not available		
26	11/2012	Stool	A3	Metallo-β-lactamase	*bla*_VIM_
27	11/2012	Stool	A5	Metallo-β-lactamase	*bla*_VIM_

A total of 35 environmental surveillance samples were collected (22 in October 2011, 13 in January 2012), none of which resulted to be positive for XDR-PA.

## Discussion

In Italy, an increase in frequency of bacteremia due to MDR-PA has been described in pediatric hematology oncology centers [1]. The outbreak we report, which occurred in children with onco-hematological diseases, is particularly worrisome, considering that not only bacterial virulence but also a state of profound impairment of immune defense contribute to both morbidity and mortality. In fact, clinical presentation of XDR-PA was severe: out of 18 infected patients, 12 had bacteremia, and 67% of them died due to XDR-PA bloodstream infection. The observed mortality rate was higher than that reported for MDR-PA bacteremia cases in children [1], but lower than that described in a nosocomial outbreak of pan-antibiotic-resistant *P. aeruginosa*[14]. A recent Spanish study suggested that XDR-PA strains are particularly prone to cause bacteremia, though it was unclear whether this invasive capacity depended on clonal traits or on other virulence determinants [24]. As expected, we observed that severe neutropenia was significantly associated with XDR-PA bacteremia, compared to other infections or colonization due to XDR-PA.

Carbapenems are one of the most effective antimicrobial agents against gram-positive and gram-negative bacteria; unfortunately pathogens resistant to this antimicrobial family continue to emerge, due to plasmid or integron-mediated carbapenemases, efflux systems, reduced porin expression and increased chromosomal cephalosporinase activity [25]. All but two XDR-PA strains isolated in this outbreak were MBL producers, due to the presence of a *bla*_VIM_ gene. The first VIM (Verona integron-encoded metallo-β-lactamase) type MBLs was reported in 1999 in Italy in a *P. aeruginosa*[26]. At present these enzymes are spread through all continents, being also associated with large outbreaks of MDR *P. aeruginosa* or *Enterobacteriaceae*[27, 28].

In our study, the genetic relationship among isolates was investigated by using PFGE analysis, which has been proven to be a reliable tool for typing *P. aeruginosa* strains [3; 13; 14; 29]. As observed in other investigations [8, 9, 29], clonal groups and XDR-PA sporadic strains were identified.

It is likely that the emergence of XDR-PA observed in our hospital was related to different mechanisms, including development of resistance in previously susceptible *P. aeruginosa* isolates, horizontal transfer of MBL genes, and patient-to-patient transmission.

Under high antimicrobial pressure, there is a risk of polyclonal emergence of multiple-antibiotic-resistant *P. aeruginosa.* Use of broad-spectrum cephalosporins and of aminoglycosides antibiotics have been documented to be independent risk factors for spread of MDR-PA [29], while exposure to fluoroquinolones has been reported to be associated with XDR-PA bacteremia [24]. Exposure to antimicrobial agents is a documented risk factor also for acquired MBL [28]. All children but two we diagnosed to be affected by XDR-PA bacteremia had been given ciprofloxacin as medical prophylaxis. Judicious choice of antimicrobials used for prophylactic courses or empirical treatment should be emphasized in order to prevent *de novo* emergence of XDR-PA strains.

Asymptomatic carriers of MDR-PA play an important role in long-term in-hospital transmission [30]. Active surveillance for identification of intestinal carriers of MDR gram-negative bacteria at hospital admission, followed by adoption of contact precaution and isolation has been recommended as a measure for limiting the risk of inter-personal transmission [31].

## Conclusions

In this outbreak, the transmission chain starting from the first patient, who was diagnosed in March 2011 and whose XDR-PA isolate belonged to genotype A1, has involved a total of nine patients, over a 10-month period. The number of patients in other clusters decreased over time, with six patients for genotype A4, and two cases for genotype A3 and A5. No secondary cases were observed for four sporadic genotypes. All wards of the onco-hematological Department were involved; transmission could have occurred through hands of health care workers, or use of non-critical medical equipments such as phonendoscopes. Optimal adherence to contact precautions is essential to limit the risk of health care associated transmission of MDR and XDR microorganisms. Since the implementation of active surveillance aimed at identifying intestinal carriers along with the adoption of contact precautions, isolation or cohorting of XDR-PA carriers, incidence of XDR-PA infections was 2 times lower than that observed prior to implementation. These findings confirm that adoption of control measures targeting intestinal carriers should be undertaken in limiting in-hospital transmission of XDR-PA strains, protecting more vulnerable patients, such as severely neutropenic children, from developing clinical infections, including bacteremia. Italy is one of the European Countries with the highest proportion of multidrug-resistant and carbapenemase-producing *Enterobacteriaceae*[32]. Active tracing of fecal carriage should be recommended for high-risk hospitalized children.

## Authors’ original submitted files for images

Below are the links to the authors’ original submitted files for images. Authors’ original file for figure 1 Authors’ original file for figure 2

## Abbreviations

XDR-PA: Extensively drug-resistant *Pseudomonas aeruginosa*
PCR: Polymerase-chain-reaction
PFGE: Pulsed-field gel electrophoresis
VIM: Verona integron-encoded metallo-β-lactamase
MDR-PA: Multi-drug-resistant *P. aeruginosa*
XDR: Extensively drug-resistant
HAI: Healthcare-associated infections
ICT: Infection Control Team
MDR: Multi-drug-resistant
EUCAST: European Committee on Antimicrobial Susceptibility Testing
KPC: *Klebsiella pneumoniae* carbapenemase
MBL: Metallo-β-lactamase
OXA: Oxacillinases
UPGMA: Unweighted pair group method with arithmetic means
WBC: White blood cells
HSCT: Hematopoietic stem cell transplantation.
