# Supplementary figures and images for: An outbreak of extremely drug-resistant Pseudomonas aeruginosain a tertiary care pediatric hospital in Italy
Source: BMC Infect Dis. 2014 Sep 10;14:494. doi: 10.1186/1471-2334-14-494 (PMC4167521; doi:10.1186/1471-2334-14-494)

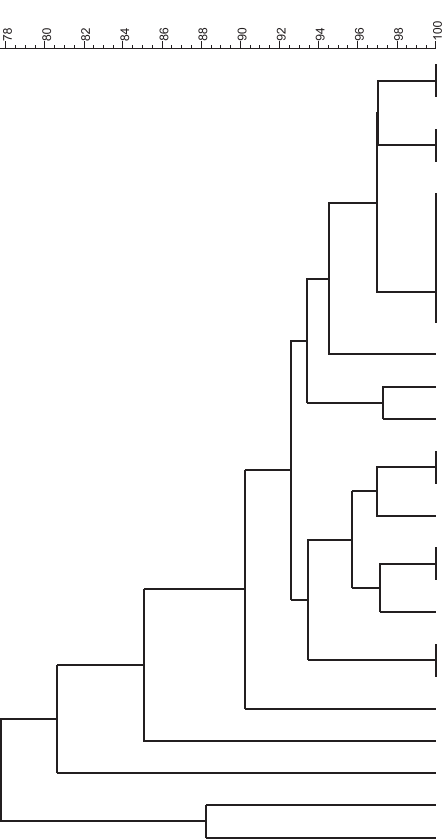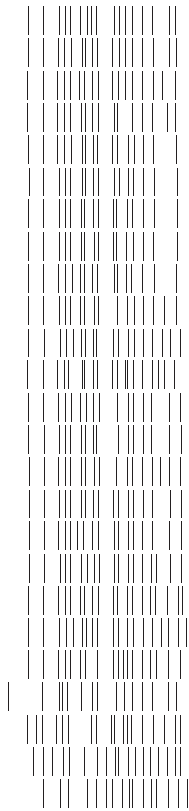

| PFGE | Patient ID |
|------|------------|
| A1   | 6          |
| A1   | 9          |
| A1   | 12         |
| A1   | 13         |
| A1   | 1          |
| A1   | 2          |
| A1   | 3          |
| A1   | 4          |
| A1   | 7          |
| A2   | 11         |
| A3   | 26         |
| A3   | 14         |
| A4   | 22         |
| A4   | 24         |
| A4   | 15         |
| A4   | 17         |
| A4   | 18         |
| A4   | 16         |
| A5   | 20         |
| A5   | 27         |
| A6   | 19         |
| B    | 10         |
| C    | 8          |
| D    | 21         |
| E    | 23         |

Supplement: Supplementary file 1 — Authors’ original file for figure 1 [file 12879_2014_3796_MOESM1_ESM.pdf]
